# The Prognostic Role of Postablative Non-Stimulated Thyroglobulin in Differentiated Thyroid Cancer

**DOI:** 10.3390/cancers13020310

**Published:** 2021-01-15

**Authors:** Szabina Szujo, Laszlo Bajnok, Beata Bodis, Zsuzsanna Nagy, Orsolya Nemes, Karoly Rucz, Emese Mezosi

**Affiliations:** 1 Ist Department of Medicine, Medical School, University of Pecs, 13 Ifjusag, H-7624 Pecs, Hungary; szujo.szabina@pte.hu (S.S.); bajnok.laszlo@pte.hu (L.B.); bodis.beata@pte.hu (B.B.); nemes.orsolya@pte.hu (O.N.); klinika@somogy.hu (K.R.); 2 IInd Department of Medicine and Nephrological Center, Medical School, University of Pecs, 1 Pacsirta, H-7624 Pecs, Hungary; zsuzsanna.nagy@aok.pte.hu; 3 Szentagothai Research Centre, University of Pecs, 20 Ifjusag, H-7624 Pecs, Hungary

**Keywords:** differentiated thyroid cancer, papillary thyroid cancer, follicular thyroid cancer, thyroglobulin, prognostic factor

## Abstract

**Simple Summary:**

In the management of patients with differentiated thyroid cancer, thyroglobulin (Tg) is used as a tumor marker to predict residual disease. After surgery, the presence or absence of persistent disease and the risk for recurrent disease should be assessed. Risk categories may be changed during the course of disease; the reclassification of patients influences the management of the disease and the intensity of follow-up. The diagnostic and prognostic roles of postoperative stimulated and one-year postablative non-stimulated Tg was evaluated. The individual lowest and highest non-stimulated Tg values during the entire follow-up were also assessed. Non-stimulated Tg values had excellent diagnostic accuracy in predicting structural disease, and the risk classification based on these was significantly more accurate regarding outcome than that based on the postoperative stimulated Tg. Analysis of the lowest and highest Tg values highlighted that a patient’s risk category can be revised based on a single Tg measurement.

**Abstract:**

Thyroglobulin (Tg) is the most important tumor marker in differentiated thyroid cancer (DTC). The aim of this study was to assess the diagnostic and prognostic roles of postoperative stimulated and postablative lowest, highest, and one-year non-stimulated Tg values obtained during the follow-up of patients with DTC. In this retrospective study, 222 radioiodine-treated, anti-thyroglobulin antibody (TgAb)-negative DTC patients having at least 9 months’ follow-up time were included (172 papillary and 50 follicular cancers; median age: 48 (from 15 to 91) years; female–male ratio: 158/64; median (quartiles) follow-up time: 54 (22–97) months). The 2015 American Thyroid Association guidelines were applied as criteria of the therapeutic response. Postoperative stimulated Tg values had significantly lower diagnostic accuracy than any of the non-stimulated postablative Tg values. One-year non-stimulated Tg had excellent prognostic value for structural disease: a cut-off value of 0.85 ng/mL had an 88.1% diagnostic accuracy. If the Tg value did not decrease below 0.75 ng/mL at any time during follow-up, the risk of residual disease was 25 times higher. The highest non-stimulated Tg during follow-up was the best predictor of residual disease (e.g., a Tg value exceeding 7.7 ng/mL indicated a 30-fold increase in risk). Non-stimulated Tg values measured during follow-up have excellent diagnostic accuracy to predict structural disease in DTC patients. The risk classification of a patient can safely be modified based on even a single Tg measurement.

## 1. Introduction

Thyroglobulin (Tg) is the most important tumor marker in differentiated thyroid cancer (DTC), and its role in the assessment of the therapeutic response is obvious [1,2]. However, there are limitations to its application, the most important of them being the presence of anti-thyroglobulin antibody (TgAb), affecting approximately 30% of DTC patients. In these cases, Tg is usually not measurable, or if measurable, it cannot be relied upon [3]. The change in the antibody titer may provide information about the extent of the residual tumor in TgAb-positive patients [4]. Earlier, the lower sensitivity of first-generation Tg assays and the inaccuracy of measurements in lower ranges were also limiting factors regarding the use of Tg as a tumor marker [5]. Therefore, for many years, the determination of stimulated Tg was suggested during follow-up. Stimulated Tg was determined using levothyroxine withdrawal or recombinant human thyroid-stimulating hormone (rhTSH) stimulation 9–12 months after primary care (usually total thyroidectomy and radioiodine (RAI) ablation) [6,7]. Recently, several reports have confirmed that the adequate sensitivity of second-generation Tg assays eliminates the need for the determination of stimulated Tg in the majority of cases [5,8,9,10,11]. This is further supported by the definitions of the therapeutic response in the latest guidelines using both on-thyroxine (non-stimulated) and stimulated Tg cut-off values and considering them equivalent [12]. With the development of Tg assays, it has also become clear that many patients do not have a recurrent but rather a persistent disease that has temporarily decreased below the limit of detection in imaging and functional tests [13].

Following surgery, Tg values are influenced by the mass of the remaining normal thyroid tissue and of the tumor, as well as the mode and duration of stimulation. Many research groups have studied the prognostic role of stimulated Tg after surgery and before RAI ablation [13,14,15,16,17,18,19,20,21,22,23,24,25,26,27,28,29]. Recently, the routine use of RAI treatment has become questionable, which has also raised the question of whether the stimulated Tg value can help in making decisions about the necessity of RAI treatment. Although the answer to this question is generally no, the prognostic role of the postoperative stimulated Tg value is found to be unanimously positive. At the same time, there is no agreement on the Tg cut-off values, partly because these fundamentally depend on the risk groups to which the patients belong. A wide range of cut-off values (i.e., between 2 and 50 ng/mL) have been analyzed in the literature. Furthermore, some researchers arbitrarily selected a special value which was already available in the literature, while others determined the optimal diagnostic accuracy by receiver operating characteristic (ROC) analyses of their own patient population. In a meta-analysis including nearly 4000 patients, the evaluated 15 studies were heterogeneous. The mean sensitivity and area under the curve (AUC) values in ROC analyses were 76.1% and between 0.84 and 0.89, respectively. Although the mean specificity was found to be 85.6%, the positive predictive value (PPV) of Tg above 10 ng/mL was only 47% [13].

The diagnostic role of postablative non-stimulated Tg has been studied by only a few groups [30,31,32]. In the work of Rosario et al., the postablative non-stimulated one-year Tg with a 1.8 ng/mL optimal cut-off value provided 72.7% sensitivity, 83.4% specificity, and 95.4% negative predictive value (NPV) for structural disease in medium-to-high-risk patients [32]. Others compared the diagnostic roles of non-stimulated and stimulated Tg values [10]. A meta-analysis of nine studies confirmed that the NPV of non-stimulated Tg values is very good (97%), recommending a stimulation test only in cases when the non-stimulated Tg values are in the measurable range [10]. Italian authors investigated the predictive role of on-thyroxine Tg and its doubling time in patients who were also investigated by positron emission tomography/computed tomography (PET/CT). A Tg value above 5.5 ng/mL and a Tg doubling time of less than one year were independent predictors of a positive PET/CT finding. The diagnostic accuracy of 18 F-fluorodeoxyglucose (^18^F-FDG) PET/CT was significantly better if this imaging method was used only in these selected patients [33].

The aim of this study was to assess the diagnostic and prognostic value of Tg at different time points in the follow-up of patients with DTC: (I) stimulated Tg after surgery, immediately before the first RAI treatment; (II) on-thyroxine Tg 9 to 12 months after RAI treatment; and the (III) lowest and (IV) highest non-stimulated Tg following RAI treatment in the entire duration of follow-up.

## 2. Results

Between 1 January 2005 and 30 June 2018, 542 patients with DTC were treated at the 1st Department of Internal Medicine, University of Pécs, Hungary. Inclusion criteria were the following: (i) availability of stimulated Tg values after surgery, before the first RAI treatment; (ii) negative TgAb; and (iii) at least 9 months of follow-up time. Inclusion criteria were met by 222 patients; 45 patients did not receive RAI therapy, 29 patients had previously received RAI treatment, 11 patients did not have postoperative Tg values, 132 patients were TgAb-positive, 58 patients had been followed in other institutions, and the follow-up time was short in 49 cases.

The patients’ baseline characteristics are summarized in Table 1. The ratio of papillary (PTC) and follicular cancers (FTC) was 77%/23%. At the time of diagnosis, FTC patients were significantly older (*p* < 0.001) and were diagnosed at significantly more advanced TNM classification of malignant tumors (TNM) stages than PTC patients. For assessment of the therapeutic response, the criteria of the 2015 American Thyroid Association (ATA) guidelines were applied [12]. The median follow-up time was 54 months (quartile: 22–97 months).

Disease-specific mortality was 4.1%. The response to therapy is shown in Table 2. At the end of follow-up, less than two-thirds of the patients were tumor free, according to ATA guidelines. Therapeutic results did not differ between PTC and FTC patients (*p* = 0.569), but treatment results were significantly worse in initially advanced tumor stages, that is, T3 or T4, N1, and M1 (*p* < 0.01).

In 204 cases, TSH stimulation was done with L-thyroxine withdrawal, resulting in a median (quartiles) TSH of 63.7 (31.2–96.5) mIU/L; in 17 patients, rhTSH was used for stimulation. During the 9–12 months of control, the median TSH was 0.12 (0.02–1.54) mIU/L, while at the end of follow-up it was 0.41 (0.06–1.72) mIU/L.

Thyroglobulin values are shown in Table 3.

There was no significant difference in any of the Tg values between papillary and follicular tumor cases. Patients with metastatic (N1 and/or M1) or T4 disease had higher Tg values at every time point; however, the lowest and highest Tg values were already significantly elevated in T3 cases compared to lower T stages.

There were significant differences in Tg values with respect to subsequent therapeutic responses (Table 4).

Even Tg values of later uncertain responders were remarkably higher compared to tumor-free cases at every time point.

**Figure 1:**
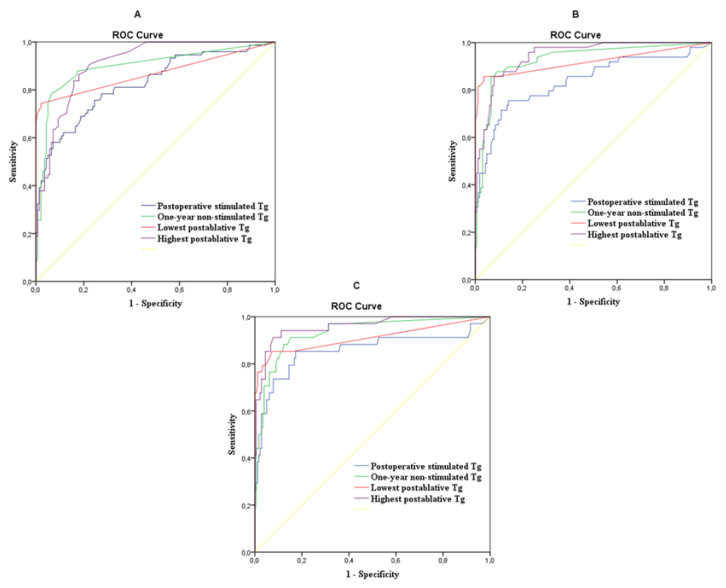
The diagnostic benefit of thyroglobulin values in the prediction of a therapeutic response during ROC analyses. (**A**) Tumor free versus uncertain + incomplete biochemical response + structural disease at the end of follow-up. (**B**) Tumor free + uncertain versus incomplete biochemical response + structural disease at the end of follow-up. (**C**) Tumor free + uncertain + incomplete biochemical response versus structural disease at the end of follow-up.

ROC analyses to predict the therapeutic response at the end of follow-up were used in the following three comparisons: (i) tumor-free patients with all others, (ii) tumor-free + uncertain responders with an incomplete biochemical response + structural disease group, and (iii) the structural disease group with all other subgroups (Figure 1 and Table 5).

AUC values measured during ROC analyses had good, occasionally excellent, diagnostic values (*p* < 0.001 in each case). If the aim was to differentiate tumor-free patients, the optimum cut-off value of postoperative stimulated Tg (20.1 ng/mL) gave lower sensitivity, specificity, and diagnostic accuracy than one-year non-stimulated Tg or the lowest Tg (0.45 and <0.1 ng/mL, respectively). In the latter case, the high specificity of 97.8% and the PPV of 97.1% should be highlighted.

If the tumor-free patients + uncertain responders were compared with patients with an incomplete biochemical response and structural disease outcomes, the prognostic roles of one-year, the lowest, and the highest Tg values were significantly better than those of postoperative Tg values (*p* = 0.004, *p* = 0.016, and *p* = 0.002).

The best AUC values were obtained in the prediction of structural disease. In this respect, the highest non-stimulated Tg value provided the greatest diagnostic benefit. If the value was above 7.7 ng/mL, residual disease could be predicted with more than 90% sensitivity, specificity, and diagnostic accuracy. The optimal cut-off value of the lowest Tg was 0.75 ng/mL, which also provided high specificity and PPV (92.7% and 92.1%, respectively), but no significant difference was found compared to the diagnostic value of postoperative Tg. The cut-off values were different depending on what the diagnostic purpose was: diagnosing a tumor-free state or finding a structural disease.

For a practical point of view, the relative risks for structural disease were also calculated using optimal cut-off values determined by ROC analysis (Table 6).

Above these thresholds, the risk of having residual cancer at the end of follow-up varied between approximately 15- and 30-fold. Based on the ROC curve, the relative risk could be calculated for all Tg values. For example, in the case of a postoperative Tg of 15 ng/mL, the relative risk for structural disease would be 7.41.

In addition to Tg values, we also examined the possible prognostic role of the Tg/TSH ratio. The AUC of the Tg/TSH ROC curve compared to stimulated Tg with respect to structural disease was lower (AUC: 0.779 versus 0.857); similarly, the prognostic significance of the one-year Tg/TSH ratio was worse than that of on-thyroxine Tg (AUC: 0.801 versus 0.933).

## 3. Discussion

In our present work, in order to predict the subsequent therapeutic response, postoperative stimulated, one-year postablative non-stimulated, as well as the lowest and highest Tg values during a median of 54 months’ follow-up were evaluated in 222 DTC patients. Patients underwent total or near-total thyroidectomy and at least one RAI treatment. According to definitions of the 2015 ATA guidelines, at the end of follow-up, only 64% of the patients were tumor free.

To the best of our knowledge, the prognostic significance of the lowest and highest Tg measured during follow-up has not been studied previously. The most important advantage of their introduction is a possible change in the patient’s risk category based on a single Tg measurement. In the determination of incomplete biochemical and structural disease outcomes, the prognostic roles of the lowest and highest Tg values were significantly better than those of postoperative Tg.

Thyroglobulin values at all times correlated well with the baseline tumor stage and the therapeutic response evaluated at the end of follow-up. The AUC values obtained in the ROC analysis were at least good (>0.8) and in half of the cases they were excellent (>0.9). The highest non-stimulated Tg measured during follow-up had the highest AUC value (AUC = 0.958) to identify structural disease. Non-stimulated Tg above 7.7 ng/mL indicated the presence of residual disease at the end of follow-up, with 91.2% sensitivity, 92.2% specificity, 91.7% diagnostic accuracy, and 30.26-fold higher relative risk compared to patients with non-stimulated Tg below 7.7 ng/mL. The prognostic value of postoperative stimulated Tg was lower than that of any of non-stimulated Tg values measured during follow-up. ROC curves of one-year, the lowest, and the highest Tg values did not differ significantly from each other.

The prognostic role of postoperative Tg has been studied in numerous publications [13,14,15,16,17,18,19,20,21,22,23,24,25,26,27,28,29,34]. Although the studies differed in the risk classification of patients and in the method of cut-off value selection, the authors found that the Tg value is an important prognostic factor. In our study, all risk groups were represented as a non-selected patient population and were evaluated in this way. The optimal cut-off values determined by our ROC analysis are consistent with the literature. The Tg cut-off value varies depending on the purpose, the selection of definitely tumor-free patients, or the detection of those with structural disease (20.1 ng/mL vs. 34.6 ng/mL in the former vs. latter cases, respectively). The current guidelines use four categories to determine the therapeutic response; both the uncertain therapeutic response and the incomplete biochemical response reflect the fact that residual cancer cannot be ruled out, but the sensitivities of imaging studies are not sufficient to detect a tiny tumor mass.

Fewer data have been published on the prognostic significance of non-stimulated Tg measured during follow-up [30,31,32]. Detection of the Tg level and ultrasound investigation during one-year control are commonly used for the patient’s reclassification, typically to a lower risk group. In our study, one-year non-stimulated Tg had excellent prognostic value (AUC: 0.933) for structural disease; using a 0.85 ng/mL cut-off value, the diagnostic accuracy was 88.1%. This low cut-off value is also consistent with the literature [32].

The fact that the prognostic significance of the Tg/TSH ratio was worse compared to the Tg value alone may suggest that TSH is only one and not the most important determinant of the Tg level.

A major advantage of our work is that a relatively large population undergoing standardized ways of diagnostic and therapeutic processes, including RAI treatments, could be evaluated. Furthermore, follow-up time intervals were long enough for reliable calculations of diagnostic characteristics, and the patient population can be considered a representative one for DTC. The strength of our study is that it provides new insight into the prognostic value of non-stimulated postablative Tg based on the therapeutic response according to the 2015 ATA guidelines, introducing the role of the lowest and highest Tg values in risk assessment and the suggestion that the reclassification of patients can be considered even based on a single Tg measurement.

### Limitations

Inter-assay variabilities may limit generalization of the cut-off values we identified. Our patient population could be biased in the direction of more advanced diseases, since everyone underwent RAI treatment. However, during this time interval, RAI ablation after surgery was routinely applied in our patients; therefore, they can be considered as representative DTC cases.

## 4. Materials and Methods

### 4.1. TSH, Tg, and TgAb Measurements

Electrochemiluminescence assays were used for the measurement of TSH (Elecsys^®^ TSH assay, Roche Diagnostics, Mannheim, Germany, measuring range: 0.005–100 µIU/mL), Tg (Elecsys^®^ TG assay from 2005 to 2014, Roche Diagnostics, Mannheim, Germany, measuring range: 0.1–1000 ng/mL; Elecsys^®^ TG II assay from 2014, Roche Diagnostics, Mannheim, Germany, measuring range: 0.04–500 ng/mL), and TgAb (Elecsys^®^ anti-TG assay, Roche Diagnostics, Mannheim, Germany, measuring range: 10.0–4000 IU/mL). TgAb determination was performed together with postoperative stimulated Tg, prior to RAI treatment. Non-stimulated Tg levels were measured at least 2 months after RAI treatment.

TSH and Tg measurements were done every 3 months in the first year, every 6 months between 1 and 3 years, and then yearly, except if the progression of the disease required more frequent visits. In individual cases, the lowest and highest Tg values were chosen from all the available Tg values.

### 4.2. Statistical Analysis

Statistical evaluation was performed using the Statistical Package for Social Sciences (SPSS, Chicago, IL, USA, Version 24.0). The distribution of the data was verified by the Kolmogorov–Smirnov test. In the case of parameters with non-normal distribution, the data were given in median and quartiles. The Kruskal–Wallis test was used to compare groups. The diagnostic role of Tg values was assessed by calculation of the true positive rate (TPR), true negative rate (TNR), PPV, NPV, and diagnostic accuracy (DA), as well as by ROC analysis. The optimum sensitivity and specificity were defined by Youden statistics. Comparison of ROC curves was done using the MedCalc statistical system according to DeLong et al. [35]. We considered *p* < 0.05 to be significant for all analyses.

## 5. Conclusions

In conclusion, we can confirm that using sensitive Tg assays, the follow-up of DTC patients has become simpler, while remaining accurate. The prognostic value of the postoperative stimulated Tg concentration is lower than that of postablative non-stimulated concentration. Non-stimulated Tg values measured during follow-up have excellent diagnostic accuracy to predict structural disease in DTC patients. The most important advantage of the introduction of the lowest and highest Tg values is the possibility to change the patient’s risk category based on a single Tg measurement.

## Figures and Tables

**Table 1:** Patients’ baseline characteristics (*n* = 222).

Parameter	*n* (%)		
	All	Papillary	Follicular
	*n* = 222	*n* = 172 (77)	*n* = 50 (23)
Age (years) *			
Median (min–max)	48 (15–91)	44 (15–82)	55 (19–91)
Sex			
Female	158 (71)	120 (70)	38 (76)
Male	64 (29)	52 (30)	12 (24)
Histology			
Papillary	-	172	-
Classical variant	-	134 (78)	-
Follicular variant	-	29 (17)	-
Sclerosing variant	-	3 (2)	-
Tall-cell variant	-	2 (1)	-
Trabecular variant	-	4 (2)	-
Follicular	-	-	50
Classical variant	-	-	41 (82)
Hurthle cell variant	-	-	7 (14)
Insular variant	-	-	1 (2)
Trabecular variant	-	-	1 (2)
T stage *			
T1	77 (35)	69 (41)	8 (16)
T2	51 (23)	31 (18)	20 (40)
T3	69 (30)	54 (32)	15 (30)
T4	22 (10)	15 (9)	7 (14)
N stage *			
N0	151 (68)	107 (62)	44 (88)
N1	71 (32)	65 (38)	6 (12)
M stage *			
M0	207 (93)	164 (95)	43 (86)
M1	15 (7)	8 (5)	7 (14)

* *p* < 0.05 if papillary cancers were compared to follicular ones.

**Table 2:** Therapeutic response rates (%) according to histological types and tumor stages and *p*-values of comparisons of subgroups (in Kruskal–Wallis tests).

**Histological Types and Tumor Stages**													
**Therapeutic Response**			**All**	**PTC**	**FTC**	**T1**	**T2**	**T3**	**T4**	**N0**	**N1**	**M0**	**M1**
Tumor free			64	65	62	77	73	54	36	76	39	69	0
Uncertain			13	12	14	12	10	19	5	13	11	14	0
Incomplete biochemical response			7	9	2	8	2	9	14	3	16	7	7
Structural disease			16	14	22	4	16	19	46	7	34	10	94
***p*-Values of Comparisons of Subgroups**													
**PTC–FTC**		**T1–T2**	**T1–T3**		**T1–T4**	**T2–T3**		**T2–T4**	**T3–T4**		**N0–N1**	**M0–M1**	
*p*	0.569	0.440	0.007		<0.001	0.063		0.005	0.030		<0.001	<0.001	

PTC: papillary thyroid cancer; FTC: follicular thyroid cancer.

**Table 3:** Thyroglobulin values by histological types and tumor stages and *p*-values of comparisons of groups (in Kruskal–Wallis tests).

**Histological Types**								
**Thyroglobulin (ng/mL)**	**All**			**PTC**		**FTC**		
	**Median (quartiles)**							
Postoperative	15.5 (4.8–39.8)			14.8 (4.6–39.3)		17.3 (4.7–47.7)		
After 9–12 months	0.0 (0–1.1)			0.1 (0–1.2)		0 (0–0.6)		
At the end of follow-up	0 (0–0.6)			0 (0–0.6)		0 (0–0.6)		
The lowest	0 (0–0.2)			0 (0–0.3)		0 (0–0)		
The highest	0.5 (0.1–4.45)			0.55 (0.2–4.3)		0.45 (0–14.6)		
**Tumor Stages**								
**Thyroglobulin (ng/mL)**	**T1**	**T2**	**T3**	**T4**	**N0**	**N1**	**M0**	**M1**
	**Median (quartiles)**							
Postoperative	12.9	13.5	14.9	71.0	11.2	25.9	13.5	638.0
After 9–12 months	0	0	0.1	5.7	0	1.3	0	47.7
At the end of follow-up	0	0	0	3.5	0	0.7	0	22.0
The lowest	0	0	0	1.3	0	0.2	0	15.0
The highest	0.3	0.4	0.7	9.4	0.4	2.8	0.4	159.8
***p*-Values of Comparisons of Groups**								
**Thyroglobulin**	**PTC–FTC**	**T1–T2**	**T1–T3**	**T1–T4**	**T2–T3**	**T2–T4**	**T3–T4**	**N0–N1**
	***p***							
Postoperative	0.540	0.748	0.277	0.015	0.246	0.024	0.045	<0.001
After 9–12 months	0.723	0.183	0.093	0.001	0.502	0.015	0.012	<0.001
At the end of follow-up	0.481	0.408	0.008	<0.001	0.096	0.002	0.009	<0.001
The lowest	0.671	0.263	0.011	<0.001	0.161	0.001	0.006	<0.001
The highest	0.787	0.875	0.030	<0.001	0.045	<0.001	0.002	<0.001

PTC: papillary thyroid cancer; FTC: follicular thyroid cancer.

**Table 4:** Thyroglobulin values according to therapeutic responses.

Thyroglobulin (ng/mL)	Tumor Free	Uncertain	Incomplete Biochemical Response	Structural Disease
	Median (Quartiles)			
Postoperative	9.8 (3.2–19.7)	23.0 (9.7–52.1)	45.4 (16.3–60.7)	215.0 (65.0–638.0)
After 9–12 months	0 (0–0)	0.5 (0–1.0)	1.7 (1.3–5.7)	21.6 (4.3–104.4)
At the end of follow-up	0 (0–0)	0.5 (0.4–0.6)	2.0 (1.4–3.0)	19.1 (7.8–117.0)
The lowest	0 (0–0)	0.1 (0–0.4)	0.9 (0.6–1.9)	10.2 (3.0–40.0)
The highest	0.2 (0–0.5)	1.5 (0.6–2.4)	5.0 (2.0–8.8)	63.7 (11.4–1254.0)

All differences were significant between groups (Kruskal–Wallis test; *p* < 0.01) except the postoperative thyroglobulin value between uncertain and incomplete biochemical response groups.

**Table 5:** The diagnostic benefit of thyroglobulin values in the prediction of therapeutic response (by ROC analysis).

**Tumor Free vs. Uncertain + Incomplete Biochemical Response + Structural Disease at the End of Follow-Up**								
**Thyroglobulin**	**AUC**	***p*-Value**	**Cut-Off (ng/mL)**	**Sens%**	**Spec%**	**PPV%**	**NPV%**	**DA%**
Postoperative	0.825	<0.001	20.1	75.7	75.5	75.5	75.7	75.6
After 1 year	0.897	<0.001	0.45	78.4	93.5	92.3	81.2	86.0
The lowest	0.868	<0,001	<0.1	74.3	97.8	97.1	79.2	86.1
The highest	0.911	<0.001	0.85	86.4	82.0	82.8	85.8	84.2
**Tumor Free + Uncertain vs. Incomplete Biochemical Response + Structural Disease at the End of Follow-Up**								
**Thyroglobulin**	**AUC**	***p*-Value**	**Cut-Off (ng/mL)**	**Sens%**	**Spec%**	**PPV%**	**NPV%**	**DA%**
Postoperative	0.838	<0.001	41.2	71.4	89.0	86.7	75.7	80.2
After 1 year	0.928	<0.001	0.85	87.8	90.9	90.6	88.2	89.4
The lowest	0.919	<0.001	0.45	85.7	96.3	95.9	87.1	91.0
The highest	0.945	<0.001	3.35	85.7	92.1	91.6	86.6	88.9
**Tumor Free + Uncertain + Incomplete Biochemical Response vs. Structural Disease at the End of Follow-Up**								
**Thyroglobulin**	**AUC**	***p*-Value**	**Cut-Off (ng/mL)**	**Sens%**	**Spec%**	**PPV%**	**NPV%**	**DA%**
Postoperative	0.857	<0.001	34.6	85.3	82.7	83.1	84.9	84.0
After 1 year	0.933	<0.001	0.85	91.2	84.9	85.8	90.6	88.1
The lowest	0.909	<0.001	0.75	85.3	92.7	92.1	86.3	89.0
The highest	0.958	<0.001	7.7	91.2	92.2	92.1	91.3	91.7

AUC: area under the curve; PPV: positive predictive value; NPV: negative predictive value; DA: diagnostic accuracy.

**Table 6:** Relative risk of structural disease based on cut-off values of thyroglobulin determined by ROC analysis.

Thyroglobulin	Cut-Off Values (ng/mL)	Relative Risk (RR)
Postoperative	34.6	15.87
One-year non-stimulated	0.85	28.53
Lowest non-stimulated	0.75	25.18
Highest non-stimulated	7.7	30.26

## Data Availability

The data presented in this study are available on request from the corresponding author. The data are not publicly available due to ethical considerations.
